# Report from the Ready for the Next Round Thought-Leadership Roundtables on Building Resilience in Cancer Care and Control in Canada-Colorectal Cancer Canada; 2021

**DOI:** 10.3390/curroncol29030143

**Published:** 2022-03-07

**Authors:** Eliya Farah, Maria El Bizri, Radmila Day, Lavina Matai, Fred Horne, Timothy P. Hanna, David Armstrong, Susan Marlin, Olivier Jérôme, Darren R. Brenner, Winson Cheung, Laszlo Radvanyi, Eva Villalba, Natalie Leon, Chana Cohen, Karine Chalifour, Ronald Burkes, Sharlene Gill, Scott Berry, Brandon S. Sheffield, Pamela Fralick, Barry D. Stein, on behalf of Ready for the Next Round Patient Panelists

**Affiliations:** 1 Colorectal Cancer Canada, Montreal, QC H3G 1J1, Canada; mariae@colorectalcancercanada.com (M.E.B.); rbday79@gmail.com (R.D.); lavinam@colorectalcancercanada.com (L.M.); chanac@colorectalcancercanada.com (C.C.); barrys@colorectalcancercanada.com (B.D.S.); 2 School of Public Health, University of Alberta, Edmonton, AB T6G 1C9, Canada; fred@horneassociates.ca; 3 Department of Oncology, Queen’s University, Kingston, ON K7L 5P9, Canada; tim.hanna@kingstonhsc.ca (T.P.H.); scott.berry@kingstonhsc.ca (S.B.); 4 Division of Cancer Care and Epidemiology, Queen’s University, Kingston, ON K7L 5P9, Canada; 5 Farncombe Family Digestive Health Research Institute, Department of Medicine, McMaster University, Hamilton, ON L8S 4K1, Canada; armstro@mcmaster.ca; 6 Clinical Trials Ontario, Toronto, ON M5G 1M1, Canada; susan.marlin@ctontario.ca; 7 CATALIS-Clinical Trials Quebec, Montreal, QC H3C 3X6, Canada; ojerome@catalisquebec.com; 8 Department of Oncology, Cumming School of Medicine, University of Calgary, Calgary, AB T2N 4N1, Canada; darren.brenner@ucalgary.ca (D.R.B.); winson.cheung@albertahealthservices.ca (W.C.); 9 Department of Cancer Epidemiology and Prevention Research, Alberta Health Services, Cancer Control Alberta, Calgary, AB T2S 3C3, Canada; 10 Ontario Institute for Cancer Research, Toronto, ON M5G 0A3, Canada; laszlo.radvanyi@oicr.on.ca; 11 Coalition Priorité Cancer au Québec, Saint-Lambert, QC J4P 2J7, Canada; evav@coalitioncancer.com; 12 Jewish General Hospital, Montreal, QC H3T 1E2, Canada; nleon@jgh.mcgill.ca; 13 Young Adult Cancer Canada, St. John’s, NL A1A 5C5, Canada; karine@youngadultcancer.ca; 14 Mount Sinai Hospital, Toronto, ON M5G 1X5, Canada; ron.burkes@sinaihealth.ca; 15 Cancer Institute, Princess Margaret Hospital-Ontario, University Health Network, Toronto, ON M5G 1L7, Canada; 16 BC Cancer, Vancouver, BC V5Z 4E6, Canada; sgill@bccancer.bc.ca; 17 Department of Medicine, Division of Medical Oncology, Faculty of Medicine, University of British Columbia, Vancouver, BC V5Z 4E6, Canada; 18 Division of Advanced Diagnostics, William Osler Health Centre-Brampton Civic Hospital, Brampton, ON L6R 3J7, Canada; brandon.sheffield@williamoslerhs.ca; 19 Innovative Medicines Canada, Ottawa, ON K1P 6L5, Canada; pfralick@imc-mnc.ca

**Keywords:** COVID-19, colorectal cancer, resilience, patient-centricity, virtual care, clinical trials, real-time data, burnout, backlog

## Abstract

(1) Background: The COVID-19 pandemic illuminated vulnerabilities in the Canadian health care system and exposed gaps and challenges across the cancer care continuum. Canada is experiencing significant disruptions to cancer-related services, and the impact these disruptions (delays/deferrals/cancellations) have on the health care system and patients are yet to be determined. Given the potential adverse ramifications, how can Canada’s health care systems build resilience for future threats? (2) Methods: To answer this question, CCC facilitated a series of four thought-leadership roundtables, each representing the views of four different stakeholder groups: patients, physicians, health care system leaders, and researchers. (3) Results: Six themes of strength were identified to serve as a springboard for building resilience including, (1) advancing virtual care and digital health technologies to prevent future interruptions in cancer care delivery. (2) developing real-time data metrics, data sharing, and evidence-based decision-making. (3) enhancing public–private-non-profit partnerships to advance research and strengthen connections across the system. (4) advancing patient-centricity in cancer research to drive and encourage precision medicine approaches to care. (5) investing in training and hiring a robust supply of health care human resources. (6) implementing a national strategy and infrastructure to ensure inter-provincial collaborative data sharing (4). Conclusions: A resilient health care system that can respond to shocks and threats is not an emergency system; it is a robust everyday system that can respond to emergencies.

## 1. Terms of Reference

### 1.1. Purpose

The unprecedented nature of the COVID-19 pandemic and its consequential disruption to primary cancer care and control services has necessitated a logistical transformation in health care provision across hospitals, clinics, and centers worldwide. The pandemic continues to interrupt regular patient flow to health care facilities and cancer-related screening and treatment procedures have been delayed, postponed, or completely halted nationwide. A recent study by Canadian and UK researchers suggests that for every month that cancer surgery is delayed, mortality is expected to increase by about 10% or even more in some cases [1]. The COVID-19 pandemic illuminated vulnerabilities in the Canadian health care system, and exposed gaps and challenges that could be turned into opportunities. It also spawned a spontaneous effort of researchers and clinicians to break down barriers to collaboration and data sharing to solve real-time problems inflicted by the pandemic that should be continued.

The Ready for the Next Round Thought-Leadership Roundtables initiative, facilitated by Colorectal Cancer Canada (CCC), is a series of four thought-leadership roundtables, each representing the views of four different stakeholder groups: patients, physicians, health care system leaders, and researchers. The series was initiated on 21–24 September 2021 and aimed to examine multiple core areas pertaining to the COVID-19 pandemic and cancer. There were four aims to the roundtables: first, to explore and understand the impact of the pandemic on cancer control (e.g., screening) and care (e.g., treatment) in general, but more specifically colorectal cancer. Second, to foster ideas, strategies, and solutions to address current backlogs and prevent future interruptions in cancer care. Third, to pinpoint the lessons that we have learned from the pandemic that could be useful to the provision of cancer care in case similar crises occur in the future. Fourth, to find innovative strategies to strengthen our health care and research systems to build a more resilient infrastructure that can respond better to future situations. 

### 1.2. Participants

The Ready for the Next Round thought-leadership roundtable panelists included patients, patient advocates, nurse navigators, medical oncologists, gastroenterologists, pathologists, radiation oncologists, epidemiologists, and private and public health care representatives across Canada (Table 1). 

### 1.3. Target Audience

The recommendations presented here are targeted at the federal, provincial, and territorial (FPT) health care professionals and researchers as well as health policy advisors who are directly or indirectly involved in the provision of cancer prevention, care, and control services in Canada.

### 1.4. Basis of Recommendations

The recommendations put forth are based on discussions and expert informed opinions held in each of the four thought-leadership roundtables hosted by CCC. Additional information was incorporated by the authors to further expand on particular themes discussed at the conference.

## 2. Question 1. How Do We Mitigate the Impact of the COVID-19 Pandemic on the Oncology Community and the Provision of Cancer Care?

### 2.1. Recommendations

Due to the dramatic decrease in cancer screenings, visits, therapies, and surgeries observed during the COVID-19 pandemic, Canadian health care entities must now take deliberate actions to mitigate the impact of delayed diagnoses and care by:

- Increasing human resource capacity (training and hiring) in order to allow for the expansion of operating hours for diagnostic and screening endoscopies (i.e., evenings and weekends);
- Expanding diagnostic and screening endoscopy services through partnering with private clinics;
- Optimizing available health care resources. For example, opening screening and other relevant services (i.e., non-urgent surgeries and procedures) in areas with a low prevalence of COVID-19; and
- Expand systems for the sharing of health and clinical data in collaborative data-sharing networks driving health services research to build more resilience in the cancer care system.

Expanding services to evenings and weekends will increase screening capacity and provide options for people who cannot get time off work during working hours and additionally, facilitate more flexible endoscopy scheduling and navigation at the provincial level. This can be enhanced by patient navigators, who can help mitigate high cancellation rates for colonoscopies and prevent wasted resources.

In parallel to increasing capacity and screening clinic hours, we may need to start re-training staff, open more part-time positions, and ask specialists to temporarily scale back on non-urgent work such as research or teaching to focus more on clinical practice. In addition, primary care practitioners may need to become more involved in diagnostic testing and/or provide other services and consultations along the cancer care trajectory to alleviate some burden from oncologists. Primary care providers could assist with triaging patients to enable more strategic allocation of resources and services. Proactively triaging patients based on their risk status is expected to optimize access to services and provide appropriate care for the most urgent medical conditions.

*“Capacity and human resources are the issues, not only because of COVID-19 but because of what is happening with cancer care in general. We can pour money into buildings, but it is human resources we need; we need nurses, family practitioners, oncologists, surgeons.”*

—Sharlene Gill

Telehealth and digital health in oncology can be utilized during the pandemic for real-time video consultations, counselling, medication prescribing, management of long-term treatment as well as post-discharge coordination, virtual care support, wellness interventions, health education, physical activity, diet monitoring, medication adherence and cognitive fitness. These are some of the benefits offered by telehealth that will offset the impact of the pandemic on cancer patients, survivors, and caregivers.

*“Although it does not reduce the overall workload and in fact may at times increase it, telehealth reduces the numbers of patients attending the outpatient clinics and hospital visits and reduces exposure of patients and staff to COVID-19.”*

—Ron Burkes

Wellness monitoring, education, and other health interventions should also be coupled to the development of a long-term national strategy for early cancer detection and interception, leveraging Canada’s tremendous strength in cancer genomics, proteomics (serology), and imaging as well as existing infrastructure conducting population health studies (e.g., CanPath). The “Early Detection and Diagnosis of Cancer: Roadmap to the Future” blueprint from Cancer Research UK (CRUK) is a good example of a possible national strategy in this regard [2]. 

In addition, there is a need to collaborate with various stakeholders to develop pathways and prioritization frameworks to support the sharing of information and ensure equitable access to cancer-related services during periods of constrained resources. Furthermore, the use of screening program data to inform capacity planning during periods of limited resources will benefit the health care system. Efforts to support health care resilience must address inequities in access to high-quality, timely, and safe screening across the country and not exacerbate existing disparities. We need to find opportunities to provide care closer to home, especially for those living in remote locations in Canada who continue to experience reduced access to care during the pandemic.

### 2.2. Summary of Evidence

The patients at the Patient Thought-Leadership Roundtable voiced their collective frustration as the system failed to accommodate their critical conditions during the pandemic. R.M., a long-term cancer survivor from Nova Scotia, learned that her cancer had recurred on the day the pandemic was declared. Despite already being “in the system”, she had to wait three months for chemotherapy and five months to meet a surgeon virtually. Another patient, N.L., explained that the constant changes to health system operations by health care authorities put additional pressure on health care providers who had to adapt very quickly to the new norms and standards that the pandemic forcefully introduced. Cancer patients who tested positive for COVID-19 faced more uncertainty and worry because already-scheduled tests, appointments, and treatments needed to be further delayed or even cancelled [3,4]. 

Another overlooked situation during the pandemic involved patients admitted to hospitals or visiting clinics unaccompanied by a caregiver. Facing treatment decisions or being asked to make life-death decisions requires support. Additionally, having conversations over the phone in crowded hospital rooms with limited privacy was also very difficult. Furthermore, patients were left to rely on each other for emotional support without family members at their bedside. 

The COVID-19 pandemic, according to physician panelists, had a broad impact on cancer care and control services as it interrupted and delayed screening, diagnosis, and altered the way patients were treated. Shortly after the first wave in March 2020, many Canadian provincial public health agencies issued clinical guidelines with criteria for prioritization and triage of cancer patients [5]. CRC prevention measures and diagnostic tests such as endoscopies, colonoscopies, computerized tomography (CT) scans, and magnetic resonance imaging (MRI) scans dramatically declined amid the first wave of the pandemic [6]. Similarly, nonurgent surgical procedures and other forms of elective treatment were postponed or halted [6,7,8]. One panelist, Dr. Berry, agreed that the restrictions on screening programs in Ontario during the first wave of the pandemic created significant backlogs that would take a long time to remediate. Health care staffing shortages and the reallocation of health care providers to counter the surge of COVID-19 cases introduced another set of challenges that exacerbated the existing access barriers to cancer care and control services [9,10,11]. 

Dr. Sheffield described the impact of the COVID-19 pandemic on pathologists. He explained that the pandemic had a significant impact on the types of samples that were analyzed. Interruptions in screening programs caused the shift in the type of samples pathologists were analyzing [12]. Pathologists took on additional responsibilities for COVID-19 testing, which was not widely recognized. Furthermore, the same “ingredients” (equipment and chemicals) are used to test for COVID-19 that are needed to evaluate cancer samples for neoadjuvant therapy. Thus, it became the pathologist’s responsibility to secure sufficient supplies for neoadjuvant care in order to enable medical oncologists to provide patients with treatments while waiting for surgery. 

Among the health care thought-leadership roundtable panelists, Dr. Armstrong explained that the pandemic affected cancer care as well as chronic diseases that are lower priority compared to cancer. For example, patients with inflammatory bowel disease had a wait time of 18 months because endoscopy nurses were re-deployed elsewhere within the system. Dr. Armstrong mentioned that “*even before the pandemic, hospitals in Canada were running at 110% to 120% capacity. To return to former capacity levels as well as to accommodate backlogs created by the pandemic will be a challenge. We need resilience within the healthcare system, but also re-engineering of the frameworks within which the system operates”.*

Dr. Hanna emphasized that the pandemic led to significant decreases in cancer diagnosis across Canada and internationally. Consequently, a shift in cancer stage with more people diagnosed with advanced disease at the time of presentation is expected. This will significantly impact the complexity of treatments, health care system costs, and ultimately, the outcomes. Dr. Armstrong pointed out that the pandemic also exacerbated disparities within the health care system-the pre-pandemic disadvantages experienced by lower socioeconomic groups with regard to screening are now even more pronounced [13]. As screening services re-open, it will be harder to reach these groups due to system overload, which will intensify the disparities. 

In the research thought-leadership roundtable, Dr. Malagón explained that the COVID-19 pandemic caused many laboratories to shift from clinical- to epidemiological-focused research. The pandemic, according to Ms. Marlin, led to the proliferation of COVID-related multi-center studies. Many trials assessing COVID-19 prevention and treatment were initiated. Approximately six months into the pandemic, non-COVID-related trials picked up, leading to more oncology trials being initiated in 2020 compared to 2019.

Dr. Radvanyi explained that researchers adapted quickly due to the nature of their work and their ability to work remotely. The pandemic did not impact interactivity, and researchers stayed connected. They communicated and collaborated on national and international levels regarding COVID-19-related issues (i.e., how to track variants, molecular biology of the virus, etc.). In fact, communication and collaboration on national and international levels, especially regarding COVID-19-related issues (i.e., how to track variants, molecular biology of the virus, etc.), were significantly enhanced. Barriers to data sharing and multicenter research rapidly broke down to deal with the crisis. This new and more open networked approach to research and clinical trials should be sustained and expanded post-pandemic, also offering new health professional career opportunities in areas such as data management and network management [9,14].

## 3. Question 2. How Did the COVID-19 Pandemic Influence the Health Care System to Invest in Up-To-Date Information and Timely Access to Health-Related Data?

### 3.1. Recommendations

The COVID-19 pandemic highlighted the value of up-to-date information and timely access to health-related data. It is recommended that the health care system invests in:

- Development and implementation of better interoperable electronic medical record (EMR) management algorithms using common data dictionaries in order to allow timely access to data;
- Systematic collection of data across health regions to observe trends across groups;
- Publicly accessible data and research outcomes; and
- Leverage of automation strategies (i.e., artificial intelligence and machine learning) that permit faster collection, processing, and reporting of the data in real-time (to also support precision oncology efforts).

Making data available in near real-time would significantly improve quality care performance measures and highlight areas where improvements in care are needed the most. First, the Canadian health care system needs better EMR management algorithms to allow clinicians to access all relevant data and information in real-time including links to research (biomarker) data on the same patients from standard-of-care or clinical trials. Second, data should be aligned and shared both inter-provincially and nationally so that nationwide collaborative decisions can ensue, which require interoperability of health data systems. It is an excellent time to start implementing some of the proposed solutions for data collection because many large hospital systems in Alberta and Ontario are already moving toward implementing common software platforms in their main EMR system. Third, substantial funds are needed to support the digital infrastructure and human resources needed for its development. EMR systems should be cohesive, interoperable, and capable of sharing data and information between provinces, hospitals, and cancer centers. The panelists also agreed that the Canadian provincial health care system needs better electronic medical record management algorithms that allow clinicians to access all relevant data and information in real-time.

Dr. Cheung explained that many ongoing projects are investigating how to use innovative technologies to manage and organize cancer data at the point of care; however, despite efforts and numerous initiatives, bringing some of these initiatives to routine practice is still five to ten years away. 

Data collection in various provincial registries is not consistent and often inadequate and not current (e.g., Quebec). Collecting data in a more organized and systematic way will allow for quicker and more reliable ascertainment of trends and outcomes amongst various socioeconomic groups and ethnicities. Data depicting socioeconomic groups and ethnicities as well as other equity-deserving groups need to be collected if management and outcome are to be measured. 

In addition, there is a need to improve the current approach to cancer data collection and reporting. While the current registry-based approaches collect data on specific tumors, classifications, histology, molecular markers etc., rapid reporting of higher-level reports on cancer incidence and prevalence and their associated outcomes (e.g., daily reports for COVID-19 numbers) with additional key indicators would be relevant from a surveillance and impact perspective.

*“Learnings from the data collection during COVID-19 could be extended to other areas and conditions to build more resilient interoperable healthcare systems. Such systems should be built from grassroots hospital platforms that cross-talk with the central database where data are stored in a safe way and available to clinicians and researchers where and when needed.*”

—Laszlo Radvanyi

It is important to make data and research outcomes publicly accessible while still respecting patient privacy legislation. For example, providers and patients should be aware of the resources and wait times to services in their region. This way, if the wait time for a specific test or procedure in a particular center is too long, a patient would be able to identify centers with a shorter waitlist. Still, for many Canadians, this does not solve the problem, as the center with the shorter waitlist may be too far away to be feasibly accessed. In addition, publications might shift from traditional academic journals with paywalls to open-access frameworks. Dissemination of peer-reviewed information is essential for the integrity and validity of published information and research, but it could also be complemented by quicker access to relevant results (including negative as well as positive results from clinical trials and other health care interventions).

Models such as those used for the pandemic could be applied to all other aspects of health care, particularly for cancer care. Moreover, the standards and platforms for the safe (maintaining patient privacy) and efficient sharing of clinical (therapeutic), health, and genomics/multiomics data now exist such as application programming interfaces developed by the Global Alliance for Genomics and Health (GA4GH), the Canadian Distributed Infrastructure for Genomics (CanDIG) and others. Thus, there is no need to “reinvent the wheel” here. The need for an infrastructure that allows for timely access to data and federated data sharing between different institutions and research facilities was reiterated throughout the roundtables. This indicates the need for data collection, sharing, and transfer agreements and policies at a federal level, despite existing provincial data silos.

### 3.2. Summary of Evidence

The COVID-19 pandemic has highlighted the value of up-to-date information and timely access to health-related data [15]. Availability of high-quality data is of critical importance for making both health policy and clinical decisions at the provincial and the patient levels [16]. However, in Canada, health-related data collection and its infrastructure are under-funded and under-resourced. Cancer registries and surveillance are vital parts of cancer prevention and control efforts. Despite the rich data available in the Canadian cancer registries, the lengthy collection, inconsistent methodologies, and reporting process limit their utility and prohibit real-time actionable interventions and timely information [17].

Dr. Gill pointed out that clinicians can currently only describe the impact of the COVID-19 pandemic on treatment modalities (e.g., oral chemotherapy drugs vs. intravenous); however, data on the consequences of screening and treatment delays on clinical outcomes are not clear yet. At present, we lack timely or near real-time access to health data to quantify the impact of the pandemic on the health care system. These data gaps are a challenge for the health system, policy-makers, and health professionals to raise the alarm of what procedures are being missed, and which patients are falling behind. Without this information, the delivery of evidence-based and personalized cancer prevention and care is not feasible. For example, only a few provinces have reported on the impact of the COVID-19 pandemic on cancer care in their respective provinces, and there is still no form of national reporting. In contrast, in the United Kingdom (UK) and other European countries, health care authorities were able to enumerate overdue cancer-related screening and treatment procedures promptly, thus allowing for timely interventions to be implemented [8]. 

From a technological perspective, the panelists recognized the need to improve access to health records, data collection, and analysis. Dr. Armstrong used the UK National Endoscopic Database as a model. Software providers were given the requirements for endoscopy records and asked to incorporate them into their systems. They built a system that provides data in a common format that can be exported and interoperable, allowing for aggregate data analyses. The data could also be used to accredit endoscopy units for colorectal cancer screening and provide feedback regarding their performance. 

According to Mr. Horne, the problem is that resources are used to support traditional approaches instead of designing new models of care. It is essential to approach data as critical infrastructure that allows for addressing capacity and other issues. Mr. Horne further explained that in his experience as Health Minister, the perception that people do not want to share their personal information appeared unfounded. On the contrary, most patients wanted their information shared appropriately amongst appropriate providers and researchers to deliver better care. This point had a strong agreement among all panelists.

“*The important aspect of building the data collection infrastructure is its ability to facilitate application of data in decision-making. There should be a continuous loop where clinicians, patients and policy-makers have real-time access to published data as well as real-world evidence that is coming from different parts of the country*.”

—Fred Horne

The importance of timely, quality, open, and aggregated data has never been as clear as during the COVID-19 crisis. Many institutions, organizations, programs, and activities across Canada are collectively responsible for the provision of timely access to health and health-related data [16]. However, they are often siloed and loosely coordinated, even within a single province, let alone across provinces or legal jurisdictions. Timely data pertaining to COVID-19 cases, screening, immunization, mortality, and morbidity have been critical in understanding, managing, and mitigating the pandemic’s human, social, and economic effects. Real-time COVID-19 data dashboards play a cardinal role in designing short-term responses and accelerated preventive measures to put countries back on track [18]. Regarding data collection and reporting, models similar to those used for the COVID-19 dashboards could be applied to all other aspects of health care, particularly for cancer care. *“People are now used to seeing graphs and charts on the evening news. This trend should continue and apply to other aspects of the health care and cancer-related research initiatives.”—Talia Malagon. We need to seize the opportunity of this heightened societal awareness now to mobilize the nation as a whole to work cooperatively to build a more resilient cancer care system.*

In addition to some COVID-19-related research being delayed, a significant number of research projects that were needed could not be conducted in a timely manner. For example, cancer patients needed data on the impact of COVID-19 infection on their disease and their treatment as well as the impact of their treatment on their susceptibility to COVID-19 infection. Such research could not be conducted in Canada; therefore, the Canadian cancer community was obligated to gather and rely on data from other countries. 

Dr. Cheung explained that the Canadian response to the COVID-19 pandemic was largely disjointed and fragmented, with each province developing and implementing their own policies and approaches, rather than adopting a national strategy such as the ones seen in Australia, New Zealand, and Asian countries. As a result, Canadian data on the impact of COVID-19 are not as mature as in other countries with more unified systems. Dr. Burkes thought that the decentralized nature of our health care system might not have been the ideal system to tackle the pandemic. Strategies and directives on how to deal with pandemics should be national and come from the federal government, rather than provincial authorities. Again, this speaks to the need to share data on a provincial and national basis so that collaborative initiatives and decisions can be made.

Dr. Brenner explained that requirements for data linkage and transfer were challenging even before the pandemic, and movement toward remote work and data management further exacerbated the challenges. The pandemic also underscored limitations in data infrastructure, especially the timely access to data. On the positive side, the pandemic has led to novel collaborations between diverse groups. As a result, cancer research has become more connected with various stakeholders moving toward sharing data and solutions as well as approaches to common issues. Hospitals and research institutes quickly penned and agreed to data and material transfer agreements in a win–win situation for everyone and patients without the usual lengthy legal wrangling upfront. This can set the stage for future expedited agreements for the benefit of everyone in the future.

The need for access to timely data was highlighted and proliferated due to the pandemic. It has been shown in oncology that collecting and using data promptly can directly benefit patients. Having real-time integrated data allows clinicians and health systems to better evaluate the value of many new precision targeted drugs. The need for access to data in real-time will also be essential to assess pilot projects. Data collection infrastructure can then be used as a template for other centers. *“A successful pilot should be able to continue to collect and provide real-time data that are useful at the time rather than trying to re-engineer databases or do post hoc data analysis.”*—David Armstrong. On the positive side, the pandemic increased collaboration and data sharing between various research institutes/organizations and allowed for the seamless transfer of samples and research materials. As mentioned earlier, the willingness to more openly share data and collaborate must be reinforced by leveraging data sharing and application programming interfaces developed by organizations such as GA4GH, CanDIG, and others.

## 4. Question 3. Should Telehealth Be a Lasting Model of Care, or a Temporary Measure?

### 4.1. Recommendations

Telehealth should be further developed and streamlined to expand access to care for patients. This can ensure the:

- Continuity of virtual appointments and consultations where deemed appropriate;
- Adoption of telehealth triage systems and experimenting with different models to optimize for specific conditions;
- Capacity to carry out research remotely; and
- Implementation of partial decentralization of clinical trials.

The pandemic showcased the value of telehealth technology for virtual appointments and consultations-demonstrating that proper care can be given outside hospitals virtually, over the phone, or closer to the patients’ homes in the community. Since not all patients’ conditions are suitable for virtual care, a general policy could be implemented to encourage patients to visit in person every second or third virtual consultation, or more suitably on a case-by-case basis. This highlights the need for the implementation of safeguards to reduce the number of potential errors [19,20].

Telehealth services can facilitate the implementation of public health strategies during this pandemic by increasing social distancing. Telehealth is being used to screen patients who may have symptoms of COVID-19. Additionally, it allows access to primary care providers and specialists including mental and behavioral health experts who can provide coaching and support for patients managing chronic health conditions such as weight management and nutrition counselling. Telehealth can be used for physical and occupational therapy as well as to provide education and training for health care providers through peer-to-peer professional medical consultations. Telehealth infrastructure can also be employed to support cohort studies over time such as longitudinal wellness and coaching studies linked to studies of early cancer detection building on existing population cohorts (e.g., CanPath and the Canadian Longitudinal Study on Aging) and our ability to track biomarkers in participants over time.

While telehealth services offer many benefits for patients and caregivers, capacity issues as well as access issues can remain. Some patients are still facing prolonged waiting times for appointments. To remedy this, a panelist suggested that hospitals and cancer centers should develop and adopt telehealth triage systems to prioritize patients with urgent medical conditions. 

In situations where visual assessment and close inspection are necessary, virtual appointments may not be appropriate. Thus, moving forward, many aspects of virtual care should be further examined/reviewed, which will require additional planning and resources. COVID-19 showcased the value of telehealth technology for virtual appointments and consultations; however, an even bigger lesson is the value of human resources. The strain on health care system services will likely become even more apparent during future waves of the pandemic as the system faces burnout amongst health care providers.

### 4.2. Summary of Evidence 

The COVID-19 pandemic led to several quick wins for the health care system, which have impacted health care utilization in an integrated delivery network. For example, there is wider acceptance of telehealth, some medical care was delegated to alternate caregivers, various collaborations occurred between and within the organizations, capacity to carry out research remotely was highlighted, and the need for adaptive and partial decentralization of clinical trials was reinforced.

The COVID-19 pandemic showcased the value of telehealth technology for virtual appointments and consultations, which both health care providers and patients highly valued [9,21,22]. For example, C.A. from Ontario had a positive experience with virtual care. She was diagnosed with stage IV colorectal cancer at 47 in the midst of the second wave of COVID-19. However, her case was perceived as a “priority”, and she received an ultrasound and surgery within a few days. This was followed by home care and virtual calls with health care providers, which eliminated anxiety about getting ready, going to the hospital, and being exposed to COVID-19. 

On the other hand, N.L explained that both providers and patients needed to adjust to virtual visits, which was somewhat challenging at the beginning, especially since some patients do not have the appropriate technology. Others were not comfortable having a health care provider “peeking” into their homes and private spaces. 

It was acknowledged that the COVID-19 pandemic also had a significant impact on cancer patients and their family members, who often could not accompany patients for their appointments or visit them in hospitals [23]. Although virtual care eased some of the challenges, critical and sensitive discussions and decisions were difficult to make online or over the phone. This created additional stress for both patients and clinicians delivering news or providing advice.

Dr. Berry explained that even pre-pandemic, attempts to implement virtual visits to address space constraints at his center were very challenging. However, physician and staff commitment to rapid change when faced with the urgency of the pandemic facilitated a rapid transition to virtual care. 

Virtual care can relieve some space/time constraints, but it also has downsides. For example, virtual care could create a clerical burden for the clinicians and their administrative staff when scheduling follow-up laboratory tests and/or imaging investigations. 

Dr. Burkes pointed out that virtual visits often created more work for oncologists. Having a patient come to the clinic for an in-person meeting allows the patient to have lab work and other tests done simultaneously. When seen virtually, patients obtain their blood test elsewhere, and the results can end up in the hands of a number of health care providers due to the lack of formalized protocols for virtual care. Scheduling additional tests after virtual meetings also created more work for administrative assistants who followed-up with patients and centers/labs. 

Dr. Burkes pointed out that tumor boards and multidisciplinary rounds that have moved to a virtual format are much easier to attend. Still, he missed the one-on-one mini consults after the meeting where he could approach and talk to colleagues in radiology, radiation oncology, or surgery-“*We learn to appreciate the things we took for granted. Same with the conferences, attending a meeting in-person allows interaction with peers after a presentation and asking for their opinions. This is not possible with virtual meetings*.”—Ronald Burkes.

## 5. Question 4. How Can the Health Care System Address and Assuage Burnout among Health Care Workers?

### 5.1. Recommendations

As the health care system moves through the pandemic, it is essential to assuage burnout among health care workers by:

- Investing in a reserve of trained health care professionals;
- Increasing capacity for training health care professionals when needed;
- Investing in wellness programs;
- Creating a healthy environment where workers are not overworked and can relax, meditate, or simply sit in silence;
- Structure multi-disciplinary team for psychosocial support;
- Provide and compensate health care professionals with practical support while at work (i.e., transportation, social services for children, elderly, or animal care);
- Implement regular attending rotations and reduced durations of front-line shifts;
- Allow for planned vacations even during an outbreak;
- Implement strategies to reduce the stigma associated with mental illness;
- Allowing flexibility in work shifts; and
- Providing mental health support.

There have been numerous proposed recommendations and strategies to deal with the current burnout epidemic. A few have suggested that organizations need to ensure sufficient staffing through ongoing evaluation of workload including the mitigation of data entry and administrative burdens, making efforts to reduce overtime, and avoiding the deployment of staff in areas where they lacked training. The issue of burnout among health care providers is posing a significant burden on the health care system. To deal with this, Dr. Burkes suggested investing in a reserve of trained health care professionals, for example, nurses or physicians who are semi-retired and/or do not want to work full time. These health care professionals can be called upon when needed (i.e., a clinician or a nurse takes a vacation or needs a break). Similarly, the system should have the capacity to provide training to health care professionals if and when needed. For example, nurses from the medical ward can be trained to care for oncology patients with basic oncology skills. Dr. Hanna also suggested building in reserve capacity by supporting the many non-clinical academic roles that clinicians undertake, that help improve the quality of care, improve job satisfaction, and reduce burnout. For example, strongly supporting positions for clinician educators, clinician quality improvers, and clinician scientists could create multiple new partial FTE clinician positions that could be scaled to full-time clinical positions in emergencies. 

To further address and prevent burnout amongst health care providers, a few panelists suggested that health care institutions across Canada invest in wellness programs and create positions such as Chief Wellness Officers. These individuals will be responsible to create support for a resilient workforce and a healthy work–life balance among providers. Another practical suggestion was the provision of additional perioperative and operative staff (i.e., operating room nurses, anesthesiologists, etc.) for surgeons to expedite surgical timelines and address backlogs. Funds allocated to support these resources will be needed and can be used to pay for overtime work. Burnout is rampant and pervasive and should be taken into consideration. 

Health care providers should be encouraged and empowered to, under appropriate direction, make incremental changes to improve their practice and its efficiency. As the pandemic continues to pummel our health care system, it is essential to provide health care providers with incentives to encourage and motivate them. Providers must be recognized, and they must be shown that they are appreciated. It is vital for them to know that the system is willing to invest in their education, health, and well-being. *“We need to be able to allow people to make changes and get a reward out of their jobs. A position in a health care system is not just a position, but also a skill set and investment that needs to be maintained.”*—David Armstrong. 

### 5.2. Summary of Evidence

Burnout among health care workers has increased to levels that threaten to dismantle a functioning health care workforce [24,25,26]. Elevated burnout levels along with stress, exhaustion, and fatigue are anticipated to persist long after the pandemic [24]. The interruption of screening programs during the first wave and thereafter of the pandemic created backlogs and catching up on the backlog will be a herculean task for health care workers, who are already under incredible strain. Health services could reduce screening backlogs by expanding capacity [12]. However, key components of screening programs such as cytology and colposcopy require a skilled workforce that cannot expand quickly, and pushing people to work overtime is not realistic given the potential burnout [25].

The COVID-19 pandemic also caused intensive care units (ICUs) to be stretched to their capacity and beyond, making ICUs unavailable for post-operative care. As the system tries to catch up and as surgeries resume, surgeons as well as supporting staff will likely be “stretched to their limits.” The shortage of nurses is a nationwide problem (pre-pandemic, the nurse–cancer patient ratio was 1:1; now it is 1:4) likely to be exacerbated by vaccine mandates [27,28].

One panelist also described the impact of the COVID-19 pandemic on pathologists. On average, a pathologist assesses samples from 30–50 patients each day. Pre-COVID-19, 80% of the samples were benign growths that were relatively easy to assess (i.e., benign polyps) and 20% were complex tumor tissue cases. Interruptions in screening programs caused the shift in the type of samples pathologists were analyzing, which has put significant strains on pathologists that can lead to burnout. 

At the end of the line are medical oncologists who are likely to feel the impact as they take on the care of patients, post-surgery, who may have more advanced cancers due to pandemic delays. The significant changes that have resulted from the pandemic have taken a large toll on the wellness of health care workers and need to be prioritized and addressed as the health care system attempts to recover.

## 6. Question 5. How Can a ‘Patient-Centric Approach’ Be One Step toward a More Resilient, “Learning” Health Care System?

### 6.1. Recommendations 

Even with all the resources that are poured into the health care system, Canada still lags behind other developed countries in various measures from timely access to treatment, to health care records and life expectancy. One solution could involve moving toward a more patient-centric, “learning” health care system, a system where the patient perspective is integrated into health care policy decision-making. This could be established by:

- Providing patients with better timely access to innovative treatments;
- Using patient reported measures (PROMs) and quality of life metrics;
- Better understanding of patients’ expectations and experiences through Patient Reported Experience Measures (PREMs); and
- Develop models linking standard-of-care and clinical trials with PROs and RWE to develop biomarker-based precision medicine tools in an adaptive (learning) health system approach.

Similarly, patient-centric research could be established by:

- Involving patient organizations in the approval of projects and protocols;
- Implementing decentralized clinical trials; and
- Providing timely access to patient health records.

*“It is the patient-centricity that will give the health care system the resilience it needs.”*

—Pamela Fralick

Patient centricity includes timely access to patient health records, understanding the patients’ needs, and providing patients with timely access to innovative treatments. Current quality measures in hospitals are processes-focused (i.e., wait time for surgery); however, the outcome measures should also be patient-driven. This should include changes in measures of treatment success with patient-reported outcomes (PROs) and quality of life (QoL) moving to the forefront. Although PROs and QoL are increasingly accepted as valid outcome measures, they do not always align with the best clinical outcome expected for a particular condition and treatment. Hence, there is a need for frameworks enabling working with patients to determine what their expectations are, what they need, and how the system will achieve their needs. Solutions must be tailored to the patients’ needs and patients should be empowered to understand their disease and advocate for themselves as much or as little as they desire. In Canada, access to patient data still varies between regions and institutions. However, access to patient data should be shared with patients, and should also be shared on a provincial and national level under appropriate privacy, legal, and regulatory frameworks so that collaborative initiatives and decisions can be made. For example, as mentioned earlier, there is a need for inter-provincial collaborative data sharing efforts to improve patient triaging into treatment and screening services. 

Patient organizations and advocacy groups could be involved in the approval of projects and protocols to ensure that patients’ needs and preferences are met. Patients could also be consulted when developing systems and infrastructure for data collection because they can provide valuable and relevant input on interfaces for data entry and output. Patient-reported outcomes and QoL, which have already gained significant attention amongst policy-makers and funding agencies, should become an integral part of data collection. 

Similarly, patient-centered research can begin with the concept of decentralized clinical trials that will be of relevance not only for cancer research, but also for rare diseases. Decentralization can remove hospital setting limitations, allow for broader more equitable patient recruitment, increase patient diversity, and make trials more widely accessible [29]. However, regulatory agencies are currently not prepared for decentralization. For example, principal investigator oversight and data-sharing issues have yet to be worked out. Thus far, there are only a handful of decentralized trials, for example, in Alberta and Ontario.

With regard to data collection and reporting, models such as those used for the pandemic should be applied to all other aspects of health care, particularly for cancer care.

### 6.2. Summary of Evidence 

The panelists agreed that one of the steps toward a more resilient health care system is to shift the focus toward patients. 

*“Our health care system needs to be ‘humanized’, and the approach should be ‘predict and prevent’ instead of ‘wait till it’s broken and then fix it. We need to aim for wellness and health care instead of sick care.* By measuring what matters to patients, we can respond to their needs instead of having a one-size-fits-all approach based on what is convenient for the health care system. *Every patient is different, and when talking about personalized medicine, we need to think not only about treatments and drugs but health care as a whole.”—Eva Villalba. Here, again, the notion of wellness and continuity of care becomes even more relevant for developing a system for pre-emptive health care (cancer prevention) linked to earlier cancer detection. The federal government can take a leadership role in collaboration with existing infrastructure and expertise to accomplish this and revolutionize cancer care.*

Panelists discussed that the implementation of new EMR systems will provide an opportunity to collect and potentially share pertinent data including patient-reported outcomes (PROs) between jurisdictions. However, questions (i.e., logistics and legal implications) regarding data storage, sharing, and management are still to be resolved. The increasing relevance and value of real-world evidence (RWE) and PROs are further reflected in their recent acceptance in health technology assessment (HTA) as valid outcome measures when deliberating and deciding on funding recommendations [30]. PROs are becoming a part of the value-based approach to studying new molecules, and along with RWE, are becoming an important component of the therapeutic value of a given treatment.

Research approaches should be holistic and engage all stakeholders. Panelists pointed out the necessity of patient organizations providing ample support and leadership, bringing patient centricity to the forefront. Through such organizations, patients are increasingly involved, and patient-reported outcomes (PROs) are widely used in determining health care approaches. For example, it is important to bring together patient coalitions (e.g., Quebec Cancer Coalition) and patient advocacy groups (e.g., CCC) to the forefront by working with researchers and clinicians to lobby government, decision-makers, and payers to invest in building a robust health-data infrastructure utilizing existing database and application programming interfaces and adapting them for this purpose. 

Although access to medicines is important, access to healthy lifestyles is equally, if not even more important. “We can fill people up with drugs, but what we really need to do is work with patients and understand why they have a particular lifestyle and what we can do to help them make right choices; how we get people to decide whether or not they’re going to come to cancer screening.”—David Armstrong. Access to patient data varies between regions and institutions. For example, patients in Ontario generally have access to all the results of their tests, but there is no such access in Nova Scotia. Providers should also be aware of resources and wait times in their region. This way, if the wait time for a specific test or procedure in a particular center is too long, a patient would be aware that perhaps another center that is around five hours driving distance away has a shorter wait list. Patients can be empowered to decide whether to go there or wait until a spot closer to home becomes available. “Offering high-quality patient-centered care takes time because it involves not only understanding patients’ genomics but also their values, desires, and personal preferences for how they would like to be interacted with and cared for.”—Timothy Hanna.

The panelists explained that COVID-19-related restrictions led to the decentralization of clinical trials, or at least certain components of trials (i.e., online informed consent, increased remote monitoring, investigational products delivered to homes, off-site counselling, etc.) [31]. Consequently, trials became more patient-centric, with research activities brought to patients instead of patients travelling to treatment centers. When considering trial decentralization, patient preference should also be taken into consideration. Traditionally, patients are required to travel to a treatment center for follow-up and monitoring. Thus, decentralization may take away some of the advantages offered to patients by close follow-up protocols. It is important to consult patients to better understand their preferences. However, in some cases, centralization can drive the standardization and adoption of harmonized approaches at delivering cancer care and expediting clinical trials (e.g., multi-center trials) through central research ethics boards using common trial protocol templates and common consent forms. The Ontario Research Ethics Board (OCREB) is an example of how this can work to build a more resilient clinical trial system and increased patient access.

*“Our society should be mobilized to participate in clinical trials and research and realize that research and clinical trials are normal parts of cancer care. To that end, there is a need to develop unified, synergistic messaging and collaboration with the pharmaceutical industry in precision oncology trials as part of a learning, adaptive system linking multiomic biomarker diagnostics and discovery in a unified model. Clinicians and patients participating in the research need to be acknowledged and impassioned to feel they are in a single community working for everyone’s benefit.”*

—Laszlo Radvanyi

## 7. Question 6. How Can Patient Advocacy Groups and Non-For-Profit Organizations Support the Health Care System Amid a Crisis?

### 7.1. Recommendations

The Canadian health care system should collaborate with patient organizations and advocacy groups as they are considered valuable resources for:

- Psychosocial support and mental health services for patients;
- Improved clinical trial design, recruitment and retention of patients in trials;
- Healthcare and trial navigation for patients; and
- Dissemination of trusted care resources and information.

Psychosocial support within the health care system is limited. Such support can be provided by patient support groups. However, there is a lack of awareness within the public health care system and amongst health care providers about these groups and what they offer. Thus, there is a need for stronger collaboration and partnership between patient advocacy groups and the health care system. Both patient advocacy groups and the health care industry are working toward the same goal: improved quality of life and better patient outcomes. Although they have a symbiotic relationship and each has a unique approach to achieve these outcomes, sometimes their objectives overlap and are complimentary to achieving the aim at hand. 

Canadian charities and non-profit organizations are on the frontlines. Millions of people are turning to patient advocacy groups for assistance with everything from food security to mental health services. In addition, patient advocates can play an important role in medical research and clinical trials. Traditionally, the industry has turned to patient advocacy groups for help in recruiting patients to participate in clinical trials; however, patients should be included at the earliest of stages from ideation of a trial and throughout the clinical trial continuum [32].

Patient advocacy groups offer knowledge and grass-root services that help meet the health care industry’s need for patient and caregiver input, access, and data. The providers should know where and how to refer patients to these organizations based on patient needs, preferences, and circumstances.

The need for providing patients with better access to health care is clear. Patients often agree that they have excellent care once they “get through the door”. The problem is mobilizing patients through the system until they reach the door, which demonstrates a need for cancer care navigators and/or health care navigation services for patients. Cancer patients need somebody to guide them through cancer care, tell them what to expect, and what to ask. They need somebody who can accompany them through their journey and help them during transition periods (i.e., what to do at the end of chemotherapy, transition to palliative care, refer them to survivorship programs, etc.) [33,34]. There are transition services in Alberta that guide patients from one aspect of care to the next as it is during these transitional points that patients get lost. Panelists also recognized that survivorship is an essential aspect of patient care. With an increasing number of younger patients surviving cancer, topics such as fertility are becoming increasingly important. An institution in Toronto, Canada, the Princess Margaret Cancer Center, offers a survivorship referral program. However, it is only for patients followed at the center. More institutions across provinces should be encouraged to follow suit regarding transitioning services and support programs.

Many patient organizations and ancillary services are dependent on donations, fundraising, and contributions from the pharmaceutical industry. This highlights the need for collaborations with the health care system to ensure that these organizations can continue to function in times of crises and provide much-needed support and services to patients [35]. Independent funding should be reserved to support these groups as a permanent part of the health care system. 

### 7.2. Summary of Evidence

There is an increased need for support services and transition services in institutions. One patient explained that social support and other related services are crucial for the patient’s well-being [36]. This was particularly evident during the pandemic. When the health care system was overwhelmed, patient organizations played a crucial role in mitigating the impact of the pandemic on patients, survivors, and their caregivers. 

For example, a few panelists explained that when they could not bring a family member to their appointments, they were often in distress or heavily medicated, making it difficult for them to retain the information provided. This is where organizations such as Hope & Cope, Young Adult Cancer Canada, West Island Cancer Wellness Center, and other organizations significantly contributed support. Providing patients and families with information on wellness and their disease prognosis plays an essential role in improving the patients’ well-being, and ultimately, outcomes [12]. 

Non-profit health care organizations have been committed for years now to meeting the needs of the communities and populations they serve, especially the vulnerable and underserved [37]. Non-profits act as responsible advocates for community interests in legislative and policy arenas and work collaboratively with the private–public sectors to improve the quality of life among individuals. Collaboration with patient organizations may connect different patients experiencing the same issues. Peer support can create connection and empathy that will likely lead to patients feeling better and having better outcomes [38]. It was suggested that cancer patients be provided with a “cancer coach” who would provide them with a road map of their cancer journey and explain the role of different specialists they will encounter, the support they might need, and where and how to get the support needed. Knowing from the very beginning what resources are available could save patients lots of time and spare them additional stress and anxiety.

Bladder Cancer Canada and the Canadian Cancer Society (CCS) had a peer support mentorship program whereby people with the same stage, grade, or other disease features could be matched [39]. CCS’s peer support program was cancelled due to lack of funding. However, it should be encouraged to re-initiate this service in the future. As per improvement in ancillary patient services, panelists recognized that mental health management became a big challenge during the pandemic [40].

## 8. Question 7. How Can a Centralized Clinical Research System Operating at an Inter-provincial Level Be Able to Handle Future Health Care Disruptions? 

### 8.1. Recommendations

There should be an allocation of funds in the federal budget to manage future health care crises. There is also a need to secure continuous long-term funding for health-related research in Canada, instead of having various research groups compete for research grants and opportunities. 

There is a need for an infrastructure that allows for:

- Timely access to health data as well as data sharing between different institutions and research facilities;
- Federal level data collection, sharing, transfer agreements, and policies to support timely, centralized data access;
- A bureaucratic approach would be needed to establish processes and priorities; and
- Provincial research communities to be provided with incentives to collaborate on the development of a system that prospectively collect data.

### 8.2. Summary of Evidence 

Canada’s decentralized health care system was not prepared for clinical research disruptions caused by the pandemic. National guidelines were unavailable, therefore, each province, and in fact, each institution had its own set of policies in place (i.e., different plans for closing and re-opening different parts of the economy and systems). Thus, the system was not equipped to quickly mobilize its clinical and research workforce, and its response, from a scientific standpoint, was far from optimal. 

When compared to other countries, the lack of a centralized clinical research system is a disadvantage for Canada. For example, when the pandemic was declared, the UK was able to initiate multiple trials at multiple sites within a short timeframe due to its infrastructure and the unified system needed for such research. In Canada, research is disjointed and works in different silos that are funded based on projects and opportunities. The disadvantages and downfalls of such a framework became apparent during the pandemic, therefore, federal and provincial governments and policy-makers should consider making changes. 

Within the first month of the pandemic, a number of institutions across Ontario joined forces to develop material and data transfer agreements. The pandemic accelerated the implementation of many other processes changes, some that had already been planned for several years. Despite the efforts, the system was unprepared for COVID-19. There is a clear need for better-defined strategies for dealing with health care crises at a federal level (e.g., team of health care and scientific area experts that can be mobilized during emergencies; ability to immediately implement strategies and policies; allocation of funds for the best scientifically sound solutions to address health care crises). 

Research-related successes, innovations, and collaborations between organizations and stakeholders were prompted by the pandemic and have laid the groundwork for future innovation and health care system resilience. 

Overall, Canada needs a central and permanent federal entity such as the Biomedical Advanced Research and Development Authority (BARDA) in the USA, which encompasses these aforementioned elements that can be readily deployed on a national level in case of future health crises (we should include the impending post COVID pandemic tsunami of advanced cancer cases and morbidity as a health crisis). Rather than hastily conjured up groups of “would-be experts”, some with preconceived biases, this agency should incorporate standing committees of unbiased, apolitical scientific, and clinical experts familiar with Canadian systems to evaluate acute problems, develop the best scientifically sound solutions, and mobilize the best infrastructure across Canada (including public–private partnerships).

## 9. Concluding Question: How Should a Once-In-A-Century Crisis Be Met with a Commitment to Build More Resilient Health Care System Collectively?

### 9.1. Concluding Recommendations

Six themes of strength were identified to serve as a springboard for building resilience:

- Advancing virtual care and digital health technologies to prevent any future interruptions in the delivery of care and enhance home and community-based care;
- Developing real-time data metrics, data sharing, and evidence-based decision-making;
- Enhancing public–private–non-profit partnerships to advance research and strengthen connections across the system;
- Patient-centric, learning health care systems and research to drive a precision medicine;
- Investing in training and hiring of a robust supply of health care human resources; and
- Increasing investment in early cancer detection research and a need to develop a cohesive national strategy and infrastructure in this area.

These six themes should enable rapid adaptation to unprecedented change and collaboration among care centers, clinicians, patients, researchers, and industry. It is possible to create a system that does not have to scramble for personal protective equipment and ventilators during a future pandemic.

Recovering from the pandemic will require more than just catching up with backlogs and shortening wait lists. According to Dr. Armstrong, before the pandemic, the Canadian health care system was already walking the tightrope by operating at 120% capacity. Thus, moving forward, the system must be adapted to account for the setbacks and losses caused by the pandemic, but also to incorporate innovative technologies that will enable the system to be more resilient during future disruptions. There is a need for patient engagement in building frameworks to address their care. Only by consulting with patients directly will health care systems be able to determine their expectations and how the system can meet their needs. This will be achieved through collaboration, communication, and by setting and aligning agendas around patient-centered care.

The first step in building a more resilient research system is to translate some of the key learnings from this pandemic into policies. As pointed out by the panelists, standardized practices should be implemented at a federal level. A united research system that operates at the national level, similar to that seen in the UK, is needed to handle future health care disruptions and could also support innovation in Canada. Additionally, there should be funds allocated in the federal budget to manage future health care crises as well as continuous long-term funding for health-related research in Canada. This will create a stability and cohesion of purpose and research as opposed to the current practice of various research groups competing for research grants and opportunities. We must acknowledge that competition and fragmentation of groups for funding is not a practical solution to deal with acute health crises where established networks need to be rapidly mobilized, guided by unbiased, expert advisory bodies coordinating a national response free from special interests and based on the best data-driven scientific and health principles.

Panelists pointed out the importance of patient organizations in providing ample support and leadership, bringing patient centricity to the forefront. Solutions must be tailored to the patients’ needs, and patients should be empowered to understand their disease and advocate for themselves as much or as little as they desire. 

### 9.2. Summary of Evidence

The fragility of health systems has never been of greater interest—or importance—than at this moment [6]. A resilient health system should prepare for and effectively respond to crises, maintain core functions, and sustain the provision of care across hospitals and clinics amid a crisis. In other words, health systems are considered to be resilient during a crisis if they protect human life, maintain a high quality of life, produce good health outcomes, and withstand unprecedented system shocks.

Before the pandemic, there were efforts to build a more resilient health care system (e.g., lessons from SARS; Report of the Advisory Panel on Health Care Innovation published in 2015 by Dr. David Naylor). Despite understanding that system change is necessary (as highlighted by SARS), the system was fragile and unprepared for the COVID-19 pandemic. There is a clear need for better-defined strategies for dealing with health care crises at a federal level. For example, a team of health care experts that can be mobilized during emergencies, protocols that can allow for immediate implementation of strategies and policies, and allocation of reserved funds for health care crises. As above-mentioned, Canada has the wherewithal to mobilize its expertise and infrastructure through a standing federal BARDA-like entity in Canada to solve major health crises.

“Changes that are to be implemented post-pandemic should be permanent and not changed every time a new party comes into power. Public health and cancer are not political issues and need to be free of political motivations and special interests. There is a need for unique, cohesive approaches, a permanent infrastructure to deal not only with the pandemic but also any other health-related issues.”—Laszlo Radvanyi. Resilient health systems underpin a country’s capacity to detect and respond to disease threats in a timely and effective manner.

Amid a crisis, a resilient health care system should adapt to unprecedented conditions and situations and alleviate any vulnerabilities across and beyond the system [41]. Experience from previous epidemics has emphasized how a frail health care design dictates the evolution of a crisis, which in most cases ends up being poorly handled [42]. A robust health care system that can respond to shocks and threats is not an emergency system; it is an everyday sustainable system that can respond to emergencies. 

Resilience and the ability of people to adapt to change (i.e., adjusting quickly to working from home; quicker approval of trials by ethics boards) led to and demonstrated the resilience of research teams. Within the first month of the pandemic, several institutions across Ontario joined forces to develop material and data transfer agreements. The pandemic accelerated the implementation of many other processes changes—some already having been planned for several years.

## Figures and Tables

**Table 1:** Panelists.

Stakeholder Group	Name	Title	Organization	Province
Patient	Chana Cohen	Patient Support Specialist	Colorectal Cancer Canada	QC
	Cheryl-Anne Labrador-Summers	Touched by cancer	-	ON
	Gary Puppa	Touched by cancer	-	ON
	Eva Villalba	Executive Director	Coalition Priorité Cancer au Québec	QC
	Karine Chalifour	Program Director	Young Adult Cancer Canada	NL
	Natalie Leon	Nurse Navigator	Jewish General Hospital	QC
	Robin McGee	Touched by cancer	-	NS
	Manna Wescott	Touched by cancer	-	BC
Physician	Ronald Burkes	Medical Oncologist	Mount Sinai Hospital Princess/Margaret Cancer Centre/University Health Network	ON
	Sharlene Gill	Medical Oncologist	BC Cancer–Vancouver University of British Columbia	BC
	Scott Berry	Medical Oncologist, Medical Director, Cancer Centre of Southeastern Ontario	Kingston Health Science Centre Queen’s University	ON
	Winson Cheung	Medical Oncologist	Tom Baker Cancer Center University of Calgary	AB
	Brandon Sheffield	Pathologist	William Osler Health Centre-Brampton Civic Hospital	ON
Health care system	Fred Horne	Adjunct Professor	School of Public Health, University of Alberta	ON
	Kevin Wilson	VP of Population Health Quality & Research	Saskatchewan Cancer Agency	SK
	Laszlo Radvanyi	President & Scientific Director	Ontario Institute for Cancer Research	ON
	Pamela Fralick	President and CEO	Innovative Medicines Canada	ON
	David Armstrong	Chair- Gastroenterologist	National Colorectal Cancer Screening Network- McMaster University	ON
Research	Darren Brenner	Cancer Epidemiologist	University of Calgary	AB
	Olivier Jérôme	Director, Public & Patient Engagement	CATALIS- Clinical Trials Quebec	QC
	Susan Marlin	President & CEO	Clinical Trials Ontario	ON
	Talía Malagón	Epidemiologist	Gerald Bronfman Department of Oncology, McGill University	QC
	Timothy Hanna	Clinical Scientist and Radiation Oncologist	Queen’s University	ON

## Data Availability

The data presented in this study are available in this article.
